# Mitigation of salt stress in white clover (*Trifolium repens*) by *Azospirillum brasilense* and its inoculation effect

**DOI:** 10.1186/s40529-016-0160-8

**Published:** 2017-01-03

**Authors:** Muhammad Khalid, Muhammad Bilal, Danial Hassani, Hafiz M. N. Iqbal, Hang Wang, Danfeng Huang

**Affiliations:** 1 School of Agriculture and Biology, Shanghai Jiao Tong University, Shanghai, 200240 China; 2 State Key Laboratory of Microbial Metabolism, and School of Life Sciences and Biotechnology, Shanghai Jiao Tong University, Shanghai, 200240 China; 3 School of Engineering and Science, Tecnologico de Monterrey, Campus Monterrey, Ave. Eugenio Garza Sada 2501, Monterrey, N.L. 64849 Mexico; 4 Instrumental Analysis Center, Shanghai Jiao Tong University, Shanghai, 200240 China

**Keywords:** Salinity, Leaf physiology, Stem physiology, Salinity stress, K^+^/Na^+^ ratio

## Abstract

**Background:**

Salinity is one of the increasingly serious environmental problems worldwide for cultivating agricultural crops. The present study was aimed to ascertain the potential of beneficial soil bacterium *Azospirillum brasilense* to alleviate saline stress in *Trifolium repens*. Experimental plants (white clover) were grown from seeds and inoculated with or without *A. brasilense* bacterial strain supplemented with 0, 40, 80, or 120 mM NaCl into soil.

**Results:**

The growth attributes including, shoot heights, root lengths, fresh and dry weights, leaf area and chlorophyll content were significantly enhanced in *T. repens* plants grown in *A. brasilense* inoculated soil than un-inoculated controls, particularly under elevated salinity conditions (40, 80 and 120 mM NaCl). Malondialdehyde content of leaf was recorded to be declined under saline conditions. Moreover, the K^+^/Na^+^ ratio was also improved in bacterium-inoculated plants, since *A. brasilense* significantly reduced the root and shoot Na^+^ level under high salty environment.

**Conclusions:**

Results revealed that soil inoculation with *A. brasilense* could significantly promote *T. repens* growth under both non-saline and saline environments, and this study might be extended to other vegetables and crops for the germination and growth enhancement.

## Background

Soil salinity is considered to be the most brutal environmental factor which caused a reduction in plant growth and crops productivity (Allakhverdiev et al. 2000). Cultivated soils all over the world are becoming more saline due to continuing use of poor quality water for irrigation, excessive fertilization, and desertification processes (Ramadoss et al. 2013). Currently, more than 20% of the worlds (26% of Pakistan) agricultural irrigated land is affected by levels of salt that could markedly downscale the net agriculture yield (Munns and Tester 2008). Excessive salt concentration augments the Na^+^ and Cl^−^ ions level in different plants that negatively affect the plant survival by disrupting different plant metabolisms, cellular homeostasis, and uncoupling major biochemical as well as physiological processes (Mahajan and Tuteja 2005). Although both Na^+^ and Cl^−^ ions have the capability to induce many physiological and/biochemical disorders in plants, nevertheless, Cl^−^ is among the most hazardous (Tavakkoli et al. 2010).

Effective strategies for alleviating salinity stress involve developing salt-tolerant cultivars, in situ soil flushing, leaching excess soluble salts from upper to lower soil depths, reducing salt by collecting salt-accumulating aerial plant parts, and improvement of alkaline soils under cropping and leaching (Bacilio et al. 2004). Inoculation of crop with plant growth promoting bacteria (PGPB) is becoming more imperative as an alternative to mitigate abiotic stresses (i.e., salinity) since PGPB have been reported to overcome stress effects. Further, the beneficial properties of microbes under salinity has been attributed to the hydraulic conductivity, sequestering toxic Na^+^ ions, osmolyte accumulation, retaining higher stomatal conductance and photosynthetic activities (Dodd and Pérez-Alfocea 2012).

PGPB are a diverse group of bacteria that possess the remarkable capability to promote growth and yield of many crops and wild plants (de-Bashan et al. 2012). Several species of PGPB, particularly species of the genus *Azospirillum*, *Pseudomonas*, and *Arbuscular mycorrhizal* fungi can effectively alleviate salt stress in plants (Fasciglione et al. 2015). Among PGPB, *Azospirillum* spp. is the most investigated bacterium because of its potential to colonize a wide-range vegetable crops and to enhance the general plant performance under normal or stressed environment. Indeed, it has been reported that inoculation mitigated the undesirable effects of NaCl on wheat seedlings with *Azospirillum brasilense*. The benefits of *Azospirillum* inoculation have also been observed in chickpea irrigated with saline water (Hamaoui et al. 2001). These favorable effects of the *Azospirillum* strains might be attributed to the secretion of several types of phytohormones in the surroundings of the roots. These phytohormones are physiologically active and hence, potentially assist the plants to propagate in stress conditions (Fasciglione et al. 2015).

*Trifolium repens*, the white clover, has been considered an important forage crop throughout the world. *T. repens* is also deliberated as a folk medicine in India against intestinal helminthic worms, and an in vivo experimental study corroborated that its aerial shoots possess unique anti-custodial properties (Tangpu et al. 2005). Like several other forage crops, *T. repens* is susceptible to salinity stress; reducing its growth and number of root nodules, and as a consequence, nitrogen fixation, as well as soil fertility level, is compromised (Acharya et al. 2011). The present work was carried out to evaluate *A. brassilence* potential for growth promotion and salinity resistance in white clover.

## Methods

### Bacterial strain and growth conditions

Bacterial strain, *A. brasilense* was provided by “*The Leibniz Institute DSMZ*-*German Collection of Microorganisms and Cell Cultures”*. The strain was streaked in Lauria-Bertani (LB) agar plates and incubated at 30 ± 0.2 °C for 24 h. From LB agar plates, the bacterial cells were then transferred into LB-broth and cultured at 30 ± 0.2 °C under an agitation speed of 250 rpm to obtain 1.0 × 10^9^ colony-forming units (CFU)/mL.

### Seed treatment and germination experiment

The healthy and mature seeds of *T. repens* were cleaned and disinfected with 3.5% sodium hypochlorite (NaClO) solution for 1 min followed by soaking in 70% ethanol for 10 min, and finally washed with distilled water for further use. Pre-sterilized plastic pots (20 cm diameter) comprising 800 g of soil were used to germinate the seeds by watering with Hoagland’s solution (400 mL), which contained NH_4_H_2_PO_4_, 1 mM; KNO_3_, 5 mM; MgSO_4_, 0.5 mM; Fe-citrate, 60 μM; Ca(NO_3_)_2_, 0.5 mM; MnCl_2_·4H_2_O, 18 μM; H_3_BO_3_, 92 μM; CuSO_4_·5H_2_O, 0.6 μM; ZnSO_4_·7H_2_O, 1.6 μM and (NH_4_)_6_ Mo_7_O_24_·4H_2_O, 0.7 μM. After germination, Three uniform seedlings per pot were selected for continued growth and were treated with 1 mL bacterial suspension (1.0 × 10^9^ CFU/mL), per plant as inoculation treatment. The seedlings treated with 1.0 mL liquid LB medium were considered as control. For salt stress treatments, the seedlings were watered with Hoagland’s nutrient solution supplemented with 0, 40, 80, 120 mM NaCl. Three plastic pots (Three plants/pot) were used as replications for each treatment. Plants were grown under controlled conditions at 28 °C during the day and 16 °C at night, irrigated daily through dropper with deionized water to maintain the moisture approximately at 60% water holding capacity of the soil, efforts were made to protect the leaching of salt and all pots were randomly placed in a greenhouse.

### Physiological measurements

The plants were harvested after 60 days to determine physiological index and growth measurements including, leaf area and a whole number of leaves per plant. Measurement of leaf area was made through portable leaf area meter (Model YMJ-A, China). Leaf area/plant was divided by some leaves/plant to calculate the average leaf area. Once the plants were separated from pots, the growth parameters such as root and shoot lengths (cm) were measured by a ruler. Shoot fresh weight (g) was determined immediately, whereas the plant parts were oven-dried at 75 °C for 2-days after rinsing with deionized water for quantitative analysis of dry weights (g). Leaf chlorophyll content was analyzed following the reported method (Porra et al. 1989). Briefly, 80% acetone was used to pulverize fresh leaves sample and centrifuged at 4 °C for 10 min at 10,000 rpm. The clear supernatants, thus obtained, were collected and recording OD measured chlorophyll content at 645 and 663 nm. Thiobarbituric acid (TBA) protocol was adopted to probe the oxidative stress using the biomarker malondialdehyde (MDA) (Bao et al. 2009).

### Ionic analysis

Ionic analysis (Na^+^ and K^+^) was made using an atomic absorption spectrophotometer available in Instrumental analysis center, SJTU following the method described previously (Wang et al. 2007). Ice-chilled CaCl_2_ (20 mM) was used to wash the plant roots twice for 5 min to exchange cell wall bound Na^+^. Surface salts from the shoots were removed by rinsing it in deionized water and the sample parts (root, shoot) were oven dried at 70 °C for three days to calculate dry weights. Dried plant tissues were used for Na^+^ and K^+^ analysis. These ions were extracted from dried plant samples for 2 h at 90 °C in 100 mM acetic acid.

### Statistical analysis

All the data such as growth measurements, physiological index, ion contents, and ratio were analyzed and reported with standard deviations and means of three replicates. The means and standard errors were computed for each treatment, and the SE values were displayed as Y-error bars in the figures. The collected data was subjected to analysis of variance (ANOVA) and treatment means were compared by Duncan’s New Multiple Range Test (DMRT) at P < 0.05 using a computer-based statistical software package (IBM SPSS Statistics 21).

## Results

### White clover growth (shoot height and root length) improvement by A. brasilense

**Fig. 1:**
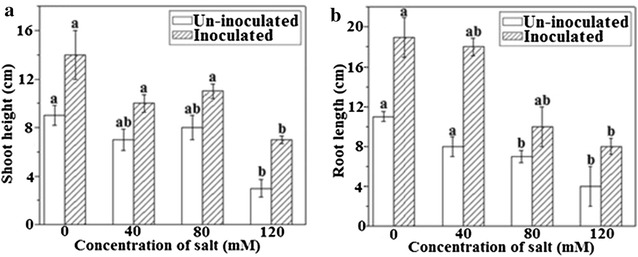
Effects of *A. brasilense* inoculation on shoot height (**A**) and root length (**B**) of *T. repens* under varying concentrations of NaCl

*Azospirillum brasilense* possessed beneficial effects on white clover cultivation by increasing its growth and decreasing the susceptibilities to different salt concentration. Under the non-saline condition, the shoot height was improved to 60.86% in comparison to media control, whereas the shoot height was significantly increased in *A. brasilense* inoculated plants than that of un-inoculated plants. The enhancement in shoot height was recorded to be 58.82, 57.8 and 70% at 40, 80, and 120 mM NaCl treatments, respectively (Fig. 1A). Compared with media control, the root length of inoculated plants was improved to 62.06% under non-saline condition. *A. brasilense* significantly increased the root length by 70.83, 58.82, and 63.63% under at experimental concentration of 40, 80, and 120 mM NaCl, respectively (Fig. 1B).

### Biomass measurement

**Fig. 2:**
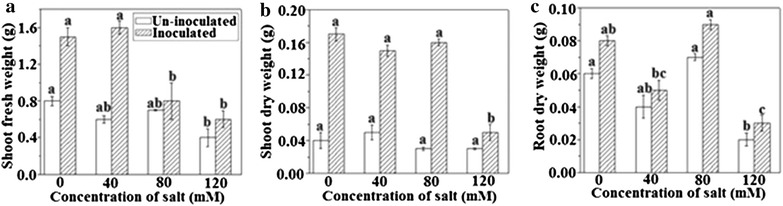
Effects of *A. brasilense* inoculation on plant growth of *T. repens* under varying concentrations of NaCl. **A** Shoot fresh weight, **B** shoot dry weight, **C** root dry weight

*Azospirillum brasilense* inoculation led to increased biomass of the white clover in non-saline conditions and also alleviated the detrimental effect of different concentration of salts (NaCl). The shoot fresh weight of inoculated white clover was found to be 2.1-folds higher in non-saline conditions as compared to similar medium control. In the same way, shoot fresh weight of experimental plants treated with 40, 80, and 120 mM NaCl were increased to 2.66, 1.16-folds and 2.33-folds (Fig. 2A). In non-saline condition, shoot dry weight was recorded to be 4.2-folds higher with respect to the control medium. While in inoculated plants, the salt stress was significantly alleviated by *A. brasilense*, and therefore, 3.0-, 5.3- and 1.6-folds improvement in shoot dry weight was observed as compared to un-inoculated ones (Fig. 2B). Inoculated plants root dry weight was assessed to be 0.57% higher in non-saline condition. Increases in dry weight of other samples were also significant and recorded to be 0.55%, 0.56 and 0.6-folds greater than un-inoculated plants at 40, 80, 120 mM NaCl concentration (Fig. 2C). In conclusion, *A. brasilense* had a significant salinity stress relieving effect on the growth of plants under all the tested concentrations of NaCl.

### Improvement in leaf number and area

**Fig. 3:**
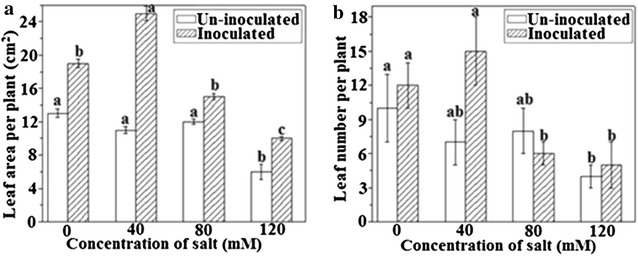
Effects of *A. brasilense* inoculation on leaf development and growth of *T. repens* under various concentrations of NaCl; **A** leaf area per plant, and **B** leaf number per plant

*Azospirillum brasilense* supported the growth of plants under abiotic (salt etc.) stress and plays a significant role in the development of leaves and chlorophyll in the absence as well as the presence of salinity stress. Leaf area of bacterial inoculated plants with no salt treatment was increased to 1.4-folds in contrast to equivalent un-inoculated samples. Stress was alleviated in the rest of treatments by increasing the number of leaves to 2.27-, 1.25- and 1.83-folds relatively at 40, 80, 120 mM NaCl concentrations, correspondingly (Fig. 3A). Enhancement in some leaves per inoculated plant under non-saline condition was 54.5%. While under NaCl treatment of 40 and 120 mM, leaf numbers were increased to 68.1 and 62.5, respectively compared with un-inoculated plants samples. Nevertheless, this trend was observed to be contradicted for the plants treated with 80 mM NaCl concentration (Fig. 3B).

### Determination of chlorophyll content

**Fig. 4:**
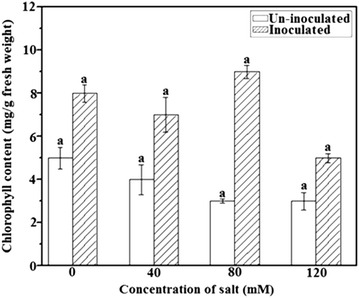
Effects of *A. brasilence* inoculation on chlorophyll content of *T. repens* under different NaCl concentrations

Besides increasing the number and area of leaves, *A. brasilense* also stimulated the amount of chlorophyll formation and supported its specific growth in bacterial inoculated plants. Leaf chlorophyll content in inoculated plants was increased to 61.5% in non-saline condition. Under appropriated saline condition, this ratio was 63.6, 75 and 0.62% grew at NaCl concentration of 40, 80, and 120 mM, accordingly (Fig. 4).

### Measurement of leaf MDA, sodium and potassium content

**Fig. 5:**
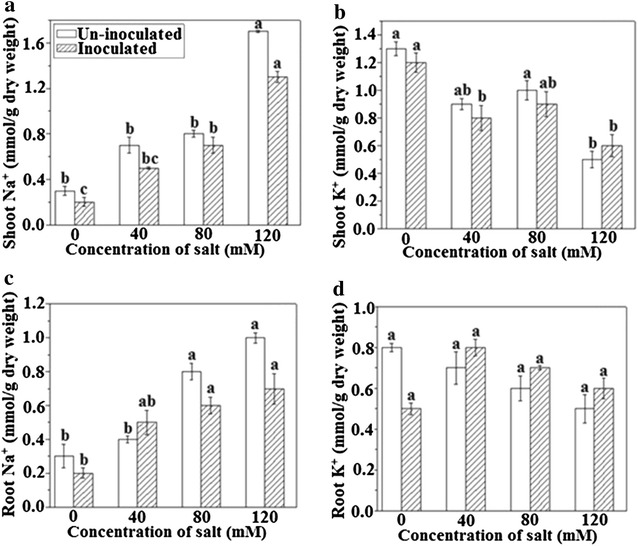
Effects of *A. brasilence* inoculation on ion-contents of *T. repens* at varying concentrations of NaCl **A** shoot Na^+^ content, **B** shoot K^+^ content, **C** root Na^+^ content, **D** root K^+^ content

Lipid peroxidation is an index of membrane damage and/or permeability, and ions leakage occurred in salinity stress environments. The results showed that MDA content in plants inoculated by *A. brasilense* decreased significantly to 64.8% in non-salinity conditions and 24.1, 20.90 and 27.5% in 40, 80, and 120 mM NaCl treatments, respectively. MDA biomarker assessment also demonstrated that *A. brasilense* could favorably regulate the oxidative stress and cell membrane integrity. The results from ion content analysis revealed that sodium content was remarkably reduced to 40% for non-saline conditions in plants treated with *A. brasilense,* whereas this ratio was 41.6, 46.66 and 43.33% increased for inoculated plants under 40, 80 and 120 mM NaCl level (Fig. 5A). Dry roots evaluation also showed that in all treatments including 0, 40, 80, 120 mM mM NaCl, the sodium content of inoculated plants decreased to 40, 55.5, 42.8 and 41.1% respectively (Fig. 5C). However, no significant reduction was recorded in potassium accumulation for all treatments (Fig. 5B, D).

## Discussion

Salt stress negatively influences the morphological, ionic, and physiological as well as biochemical characteristics in plants (Chen et al. 2013; Abbasi et al. 2015). It has been reported that increasing the salt level, reduces the osmotic potential, which results in cell dehydration, due to increased water efflux from cell (Amjad et al. 2014). Beneficial soil bacterial mediated growth enhancing attributes have been documented in enormous plant species (Gray and Smith 2005; Xie et al. 2009; Paré et al. 2011). The growth amplification of several plants, such as wheat (*Triticum aestivum*), maize (*Zea mays*), tomato (*Lycopersicon esculentum*), dwarf salt wort (*Salicornia bigelovii*), chickpea (*Cicer arietinum*), and alfalfa (*Medicago sativa*) by beneficial soil bacteria have also been observed at varying salt exposures (Bashan et al. 2000; Mayak et al. 2004; Ibragimova et al. 2006; Bano and Fatima 2009; Tiwari et al. 2011). A significant growth improvement of white clover by *A. brasilense* inoculated soil recorded in the present study was comparable with previous reports in *Arabidopsis* (Zhang et al. 2007, 2008, Zhang et al. 2010a, b; Xie et al. 2009; Paré et al. 2011). Noticeably, our findings revealed that *A. brasilense* in white clover has more active shoot growth promoting role compared to its root growth, particularly under salt stress conditions (40, 80, and 120 mM NaCl concentration). Similar results for salt preventing potentiality of *Azospirillum* strains have been documented previously by several researchers evaluated the bio-control and salt stress alleviating perspective of *A. lipoferum* in wheat crop and found that *A. lipoferum* displayed the good potential to promote the growth of wheat under saline conditions (up to 150 mM of NaCl) (Bashan and Levanony 1990). Synthesis of growth promoting substances (GPS) and adaptation to current environmental conditions may confer increased germination percentage and alleviation under prevailing salt conditions (Rueda-Puente et al. 2007; Nadeem et al. 2013). Leaf development and/or enlargement play a significant role in plant production since it is strongly associated with plant growth and biomass accumulation (Gutiérrez-Boem and Thomas 1998; Bacilio et al. 2004; Battie-Laclau et al. 2013).

A significant augmentation in leaf area and leaf number per plant was also noted in *A. brasilence* inoculated white clover plants. In another study, Han et al. (2014) reported that GB03 inoculation significantly enlarged leaf area and number per plant, and thus average leaf area in white clover. Likewise, photosynthetic pigment, leaf chlorophyll content is also an important physiological/biological attribute directly related to photosynthesis rate in plants (Ma et al. 2012). Inoculation of *A. brasilence* considerably improved the leaf chlorophyll content in white clover plant, cultivated under both non-saline as well as variable salt concentrations of 40, 80, and 120 mM NaCl. Similar to our study, Zhang et al. (2008) described that *B. subtilis* GB03 enhances photosynthetic efficiency by increasing chlorophyll content in *Arabidopsis*. In contrast, some reports highlighted that plants grown under salinity environments produced a smaller amount of chlorophyll and dry matter than those without salt exposure presumably due to chlorophyll per-oxidation (Lunde et al. 2007; Tuna et al. 2008; Barry 2009).

Soils with salinity contain an array of several cation–anion pairs (i.e., CaSO_4_, MgSO_4_, MgCl_2_, Na_2_CO_3_, and Na_2_SO_4_), with Na^+^ ions being the predominant species (Zhang et al. 2010a). Growth is impeding, a common plant response to salt stress is often related to elevated intracellular Na^+^ concentration and low K^+^/Na^+^ ratio in the plant (Zhang et al. 2010a). Researchers have reported that plants grown under saline conditions can curtail sodium toxicity by limiting Na^+^ uptake, re-directing Na^+^ from shoots to roots, and also extruding Na^+^ loadings from root cells (Tester and Davenport 2003; Munns and Tester 2008; Zhang et al. 2010a, 2011; Kronzucker and Britto 2011). In a previous study, (Zhang et al. 2010a, b) reported that *B. subtilis* significantly reduced (54%) the plant Na^+^ content in *Arabidopsis* compared with control plants by down-regulating and up-regulating *HKT1* expression in roots and shoots, respectively. In this study, soil inoculated with *A. brassilence* considerably reduced the Na^+^ level and increased K^+^ to Na^+^ ratio in both roots and shoots of the experimental plant at all levels of tested salt stresses.

## Conclusions

In conclusion, the results revealed that soil inoculation with *A. brasilense* bacterium remarkably augments plant development and biomass of the tested plant (white clover) under both salted as well as non-salted settings. The present findings offer an opportunity for the application of this beneficial soil bacterium to cultivate different plants to combat saline toxicity. Further, comprehensive investigations elucidating the mechanisms by which bacteria elicit salt tolerance would be the focus of future studies.
